# Rapid Analysis of *Listeria monocytogenes* Cell Wall Teichoic Acid Carbohydrates by ESI-MS/MS

**DOI:** 10.1371/journal.pone.0021500

**Published:** 2011-06-30

**Authors:** Marcel R. Eugster, Martin J. Loessner

**Affiliations:** Institute of Food, Nutrition and Health, ETH Zurich, Zurich, Switzerland; New England BioLabs, United States of America

## Abstract

We report the application of electrospray ionization (ESI) mass spectrometry for compositional characterization of wall teichoic acids (WTA), a major component of Gram-positive bacterial cell walls. Tandem mass spectrometry (ESI-MS/MS) of purified and chemically hydrolyzed monomeric WTA components provided sufficient information to identify WTA monomers and their specific carbohydrate constituents. A lithium matrix was used for ionization of uncharged WTA monomers, and successfully applied to analyze the WTA molecules of four *Listeria* strains differing in carbohydrate substitution on a conserved polyribitol-phosphate backbone structure. Carbohydrate residues such as *N*-acetylglucosamine or rhamnose linked to the WTA could directly be identified by ESI-MS/MS, circumventing the need for quantitative analysis by gas chromatography. The presence of a terminal *N*-acetylglucosamine residue tethered to the ribitol was confirmed using fluorescently labeled wheat-germ agglutinin. In conclusion, the mass spectrometry method described here will greatly facilitate compositional analysis and characterization of teichoic acids and similar macromolecules from diverse bacterial species, and represents a significant advance in the identification of serovar-specific carbohydrates and sugar molecules on bacteria.

## Introduction

Teichoic acids are anionic carbohydrate-containing polymers present in the cell wall of many Gram-positive bacteria, and encompass both wall teichoic acids (WTAs) as well as lipoteichoic acids (LTAs). The WTAs are covalently bound to the peptidoglycan by phosphodiester bonds between *N*-acetylmuramic acid and a special linkage unit, whereas LTAs are amphipathic molecules tethered to the cytoplasmic membrane via a glycolipid moiety [1], [2], [3], [4], [5]. In general, membrane-associated LTAs show less structural and compositional diversity than WTAs [6], [7].

Peptidoglycan-associated WTAs are highly variable in structure, and often feature species- or even strain-specific variations. Most frequently, they are comprised of polyglycerol-phosphate (GroP) or polyribitol-phosphate (RboP) chains both of which can be decorated with a variety of different sugars and/or esterified with d-alanine [8], [9]. WTAs composed of poly(RboP) are produced by important human pathogens such as *Staphylococcus aureus*, *Staphylococcus saprophyticus*, and *Listeria monocytogenes* [10].

Although their exact functions are unknown, recent data indicate important roles for WTAs in Gram-positive bacterial cells. They have been shown to assume important and essential roles in biofilm formation, in directing the cell-division machinery, as phage-binding ligands, in mediating interactions with host cells, in cation homeostasis, and as phosphate reservoir [7]. WTAs and their biosynthesis are an interesting target for development of new antibiotics, since they have been shown to contribute to host-cell binding, immune invasion, and virulence, for example in *S. aureus* [10], [11], [12]. The fact that WTA polymers are variable in structure suggests that different polymers may display distinct functions.

In *Listeria*, broad structural diversity of WTAs is brought about by variation of the glycosidic substituents attached to a polyribitol-phosphate backbone [13], [14], [15], [16], [17]. The species *L. monocytogenes* features WTAs composed of repeating RboP units mainly varying in carbohydrate components such as *N*-acetylglucosamine (GlcNAc), rhamnose (Rha), glucose (Glc), and galactose (Gal), depending on the *Listeria* strain and serovar. WTAs from strains belonging to serovar group 1/2 and 3 feature Rha (in serovar 1/2) and GlcNAc (in serovar groups 1/2 and 3) as substitution on the C2 and C4 positions of the ribitol molecule. In contrast, strains of serovars 4, 5, and 6 show a more complex WTA structure in which GlcNAc is incorporated as a part of the polyribitol chain, and may bear Glc and/or Gal substituents [16], [18]. So far, d-alanine substitution has not been reported to occur in *Listeria* WTA [15].

In order to be able to determine the role and function of WTAs in this and other bacteria more precisely, it is fundamental to obtain compositional and structural information of WTAs of wild-type strains or mutants producing altered WTA or lacking certain cell wall glycopolymers. Until now, WTA analysis has mostly been based upon laborious chemical analysis of degradation products by gas chromatography (GC) [13], and structure elucidation by ^13^C and ^1^H nuclear magnetic resonance (NMR) spectroscopy [9]. Therefore, the aim of this study was to develop a rapid and simple method for the analysis of WTA repeating units and their carbohydrate components by application of electrospray ionization tandem mass spectrometry (ESI-MS/MS), which would be applicable for investigating a larger number of bacterial strains and/or corresponding mutants.

## Results

Based on the serovar, different *Listeria* strains were chosen for the analysis of their WTA composition. *Listeria* strains EGDe, WSLC 1442, and WSLC 1485 were used as strains with GlcNAc and/or Rha substituents present on ribitol. Strain WSLC 1042 was analyzed as a member of serovar group 4, 5, and 6, where GlcNAc is an integral component of the WTA backbone chain.

### Preparation of wall teichoic acids

WTAs of strains EGDe, WSLC 1485, WSLC 1442, and WSLC 1042 isolated from purified cell walls by mild extraction with a glycine/HCl buffer resulted in water soluble, crude WTA preparations. These extracts were further purified by anion exchange chromatography on a HiTrap DEAE-Sepharose Fast Flow column. The components retained by DEAE-Sepharose Fast Flow showed high absorbance at 205 nm after elution by a NaCl gradient. Fractions were assayed for total phosphate content to identify WTA containing fractions. In all cases, WTAs eluted as a single peak in accordance with the major absorbance signal at 205 nm. Similar elution profiles were obtained for all investigated *Listeria* strains (data not shown). Phosphate-containing fractions were pooled and desalted by dialysis. For detailed compositional analysis, purified WTA polymers were hydrolyzed with hydrogen fluoride (HF) to cleave the phosphodiester bonds of the polyribitol subunits and obtain WTA monomers. The glycosidic bonds between ribitol and sugar substituents are not affected by HF treatment at 0°C [19].

### Mass spectrometry analysis of wall teichoic acid monomers

**Figure 1:**
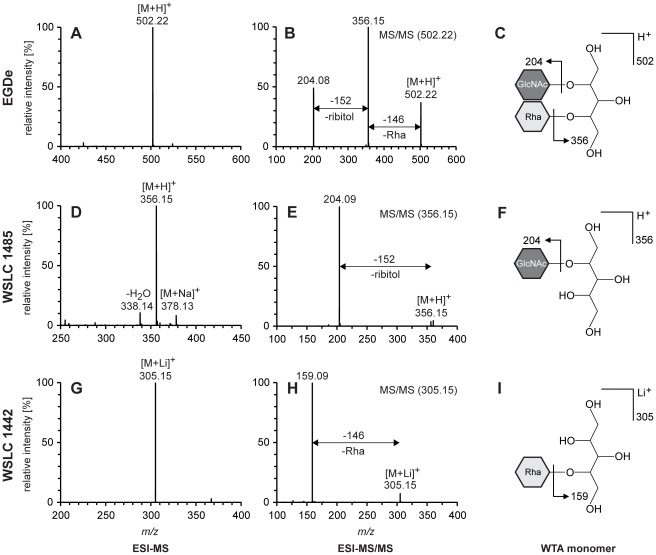
ESI-MS/MS analysis of WTA monomers of *L. monocytogenes* strains EGDe, WSLC 1485, and WSLC 1442. Left (**A, D, G**): Positive ion spectra (ESI-MS) of WTA monomers. Middle (**B, E, H**): ESI-MS/MS of the [M+H]^+^ ion (B) at *m/z* 502.22 of WTA monomer EGDe, (E) at *m/z* 356.15 of WTA monomer 1485, and the [M+Li]^+^ ion (H) at *m/z* 305.15 of WTA monomer 1442. Right (**C, F, I**): Schematic structures and side chain fragmentation of corresponding WTA monomers. Peak labels show the measured *m/z* values. Arrows denote WTA fragmentation.

**Figure 2:**
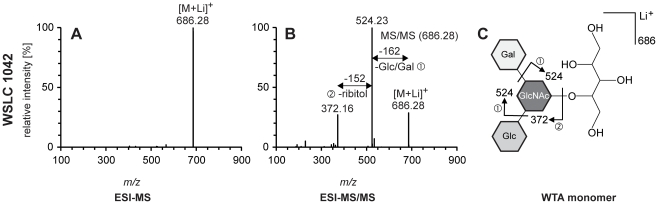
ESI-MS/MS analysis of WTAs of *Listeria* strain WSLC 1042. (**A**) Basic peak [M+Li]^+^ of the ESI-MS at *m/z* 686.28. (**B**) Product ion spectrum obtained by ESI-MS/MS for the [M+Li]^+^ ion at *m/z* 686.28. (**C**) Schematic structure and fragmentation pattern of WTA monomer of *L. monocytogenes* strain WSLC 1042. Peak labels show the measured *m/z* values. Fragment ions are indicated by arrows.

The composition of WTA monomers was determined by ESI-MS/MS. Overall, mass spectrometry findings for all WTA samples were in agreement with the previously known components of the WTAs specific for each of the different serovars. Figure 1 and Figure 2 show the mass spectra of WTA monomers, and corresponding monomeric structures as well as fragmentation patterns as the result of electrospray ionization. Compositional data were obtained by ESI-MS/MS analysis of basic peaks of monomeric WTA samples, and a synopsis of the results obtained for each of the analytes is presented in Table 1.

**Table 1:** Mass spectrometry data, carbohydrate composition of the WTA repeating units, and WGA binding properties for the bacterial strains used in this study.

*Listeria* strain	serovar	WTA repeating unit components^1^	calculated molecular weight (Da) of WTA repeating units^2^	observed mass (*m/z*) of WTA repeating units (basic peak)^3^	Binding of WGA^4^
EGDe	1/2a	Rbo, GlcNAc, Rha	501.48	502.22, [M+H]^+^	+
WSLC 1485	3a	Rbo, GlcNAc	355.34	356.15, [M+H]^+^	+
WSLC 1442	1/2a^5^	Rbo, Rha	298.29	305.15, [M+Li]^+^	−
WSLC 1042	4b	Rbo, Gal, Glc, GlcNAc	679.62	686.28, [M+Li]^+^	−

1 Gal, galactose; Glc, glucose; GlcNAc, *N*-acetylglucosamine; Rbo, ribitol; Rha, rhamnose; in serovar 4b strain WSLC 1042, GlcNAc is incorporated in the polyribitol-phosphate chain and decorated with Gal and Glc residues.

2 The calculated masses correspond to the monomeric unit of WTA, i.e., ribitol and attached carbohydrates.

3 WTAs of strains EGDe and WSLC 1485 were detected as protonated molecules, whereas WTA samples of strains WSLC 1442 and WSLC 1042 were identified as lithiated molecules.

4 +, binding; −, no binding; WGA: wheat germ agglutinin.

5 *L. monocytogenes* strain WSLC 1442 is a GlcNAc-negative serovar 1/2a mutant strain [27].

In case of WSLC 1442 and WSLC 1042, samples were prepared with lithium trifluoroacetate (Li-TFA) to be able to measure a signal in the positive ion mode, whereas WTAs from strains EGDe and WSLC 1485 were measured as protonated molecules [M+H]^+^. Each WTA type analyzed in this work revealed a characteristic MS spectrum and MS/MS fingerprint for the sample molecule. Representative spectra obtained from EGDe, WSLC 1485, and WSLC 1442 WTA are shown in Figure 1. In all cases, ribitol was substituted by GlcNAc and/or Rha residues.

WTAs of strain EGDe feature both GlcNAc and Rha residues on ribitol, resulting in a total monomeric mass [M+H]^+^ of *m/z* 502.22 (Figure 1 A). As can be seen in the corresponding ESI-MS/MS spectrum (Figure 1 B), peaks at *m/z* 356.15 and 204.08 confirm the presence of both Rha and GlcNAc. While *m/z* 356.15 is consistent with the fragment remaining upon cleavage of Rha (−146), *m/z* 204.08 represents the GlcNAc residue left after fragmentation between GlcNAc and ribitol. Note that *m/z* 204.08 may be derived from *m/z* 502.22 (loss of ribitol and rhamnose, −298), or *m/z* 356.15 (loss of ribitol, −152).

Peaks in the MS and MS/MS spectra of strain WSLC 1485 (Figure 1 D, E, and F) are nearly identical to the spectra of strain EGDe. Peak *m/z* 502 is absent, due to the lack of Rha in WTAs of strain WSLC 1485. Instead, the mass spectrum of WTA sample 1485 is dominated by a major peak at *m/z* 356.15, representing the protonated monomer [M+H]^+^ of WTA 1485 (molecular weight 355.34). In addition, ions with *m/z* 338.14 and 378.13 could be observed: the peak at *m/z* 338.14 is ascribed to the loss of a single water molecule (−18) from *m/z* 356.15, and the peak at *m/z* 378.13 corresponds to the Na^+^ adduct of the WTA 1485 monomer. In ESI-MS/MS, the [M+H]^+^ ion at *m/z* 356.15 produced *m/z* 204.09, which can be attributed to a GlcNAc residue.

For strain WSLC 1442, the component at *m/z* 305.15 characterizes a Li^+^ adduct (*m/z* +7), reflecting the monomeric WTA unit of strain WSLC 1442 (Figure 1, parts G, H and I). ESI-MS/MS analysis yielded a principal fragmentation peak at *m/z* 159.09 clearly corresponding to ribitol. These data confirm the presence of Rha in WTA 1442, which had been lost during fragmentation (−146). No GlcNAc could be detected in the spectra of strain WSLC 1442.

WTAs of strain WSLC 1042 have a more complex and unique structure: the GlcNAc residues are not only incorporated in the polyribitol phosphate chain, they also feature further modification by addition of Glc or Gal residues. Figure 2 A illustrates the data obtained by mass spectrometry analysis of WTA 1042. The prominent peak at *m/z* 686.28 corresponds to the Li^+^ adduct of the WTA monomer. After fragmentation by ESI-MS/MS, the major peak at *m/z* 524.23 (Figure 2 B) reflects elimination of the Glc or Gal substitution from the central GlcNAc, both producing the same molecular weight. The weak signal at *m/z* 372.16 likely arose from the loss of the ribitol moiety (−152), as well as a Glc or Gal residue (−162), resulting in a lithiated GlcNAc ion still carrying either Glc or Gal.

### Binding of wheat germ agglutinin to the bacterial cell surface

**Figure 3:**
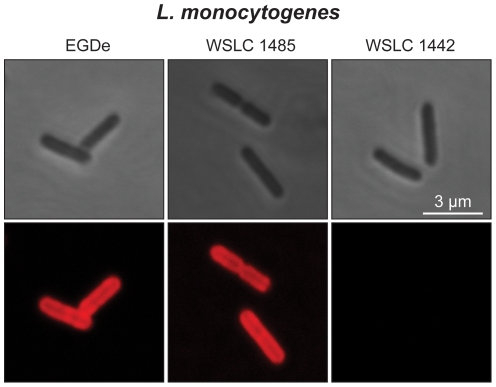
Binding of wheat germ agglutinin (WGA) to *Listeria* cells wall. *Listeria* cells of strain EGDe (left), WSLC 1485 (middle) and WSLC 1442 (right) were labeled with WGA-Alexa Fluor® 594 conjugate. WGA specifically recognizes GlcNAc residues in WTAs. Top: phase contrast images. Bottom: corresponding fluorescence microscopy (confocal) images.

The wheat germ agglutinin (WGA) lectin requires terminal GlcNAc residues on WTAs for attachment [20]. To confirm the presence of terminal GlcNAc residues on WTAs of serovar 1/2 and 3 strains, we used fluorescent WGA. Phase contrast and corresponding fluorescence images of WGA binding to strains of serovar 1/2 and 3 are depicted in Figure 3 and summarized in Table 1. WGA showed a strong association with the entire cell wall of EGDe and WSLC 1485, whereas WSLC 1442 cells were not decorated.

## Discussion

Cell wall teichoic acids represent the major surface glycopolymers of many Gram-positive bacteria. Depending on the organism, they consist of polyglycerol-phosphate or polyribitol-phosphate, decorated with d-alanine esters or mono- and di-saccharides [2]. The presence of WTAs on the bacterial cell wall affects the physiology of the bacterium and the interaction with its environment. Recently, WTAs have been increasingly studied because of their roles in biofilm formation, host cell interaction and virulence in human pathogens such as the staphylococci. WTA synthesis and linkage represent possible targets for anti-infective drugs, antibiotics, and diagnostics [7], [10], [21]. However, most of the biological functions of WTAs are still not well understood.

Structural and compositional analysis of WTAs in bacterial wild-type strains and mutants had been a challenging task, which was based mainly upon gas chromatography and ^13^C and ^1^H NMR spectroscopy. Both methods are well-established and reliable, but laborious and time-consuming. Mass spectrometry had recently been employed to study LTAs [22], [23]. We here report a rapid, sensitive and specific tandem mass spectrometric technique for the characterization of WTAs and their carbohydrate substituents, which can be applied to analyze minor compositional differences of various WTA polymers. ESI-MS requires gas-phase ions for analysis. In most cases, however, polymers can not readily be converted to gas-phase species because of the strong macromolecular structure and bonds. The creation of ion fragments by a subsequent MS/MS step is useful for determining the chemical composition of the molecule. Therefore, our aim was to elucidate the structure of WTA polymers and fragmented building blocks by ESI-MS and ESI-MS/MS.

For this purpose, WTAs extracted from *Listeria* cell walls were first purified by anion exchange chromatography, a prerequisite for quality analysis. Monitoring total phosphate content in the fractions using commercially available reagents and phosphate tests allowed rapid screening of many samples, and represents a much more convenient approach compared to previously used, time-consuming ashing procedures [24], [25]. Prior to analysis by ESI-MS and ESI-MS/MS, the WTA polymers were hydrolyzed to monomers and directly analyzed by tandem mass spectrometry in positive ion mode. Subsequent ESI-MS/MS tandem analysis permitted detection of major components based on fragment ions in the spectrum, which enabled direct identification of the individual carbohydrate moieties of WTA monomers.

As a proof of concept, four different *Listeria* strains were selected for detailed WTA analysis: EGDe, WSLC 1485, WSLC 1442 differ in carbohydrate substitution on an otherwise identical ribitol backbone structure, whereas WSLC 1042 features a different WTA type with GlcNAc embedded into the polymer chain. All WTA samples could be analyzed in positive ionization mode, and ESI spectra all yielded strong signals for the [M+H]^+^ or the [M+Li]^+^ cations. WTA samples devoid of terminal GlcNAc residues were ionized using lithium TFA as matrix component, generating Li^+^ adducts with the WTA monomers.

Results of mass spectrometry-based WTA analysis were in agreement with previously published data [13], [14], [15], [16], [26], [27]. Due to structural similarities of the different WTA types, the analytes produced similar but still characteristic ion spectra. For strain WSLC 1442, our data confirmed the unusual GlcNAc deficiency in WTA molecules. In general, GlcNAc could be directly identified by ESI-MS and ESI-MS/MS as a main component of serovar 1/2 WTAs, whereas traditional GC-MS based WTA analysis commonly fails to detect *N*-acetylated glucosamine, because of degradation of GlcNAc to glucosamine during sample preparation [15], [27]. Further, the rhamnose residues present on serovar 1/2 WTA polymers were identified as monomeric rhamnosyl moieties, rather than di-rhamnosyl components as reported previously [26], [28]. Moreover, the more complex WTA structure [14], [16] of the serovar 4 strain WSLC 1042 could also be deduced from the ESI-MS and ESI-MS/MS data. No d-alanyl substitution could be detected in the *Listeria* WTA molecules by ESI-MS/MS, which confirms previous findings [15]. However, the presence of d-alanine on *Listeria* WTA cannot be excluded since it might be lost by cleavage of the d-alanyl ester bond during sample preparation. The products encoded by the *dltABCD* genes in *B. subtilis* [29] and *S. aureus* [11] are involved in the incorporation of d-alanine not only into LTA, but also WTA. Therefore, the possibility remains that the *Listeria dlt* gene products attach d-alanine to both LTA [30] and WTA polymers.

In addition to mass spectrometry, the presence of GlcNAc in EGDe and WSLC 1485 WTA polymers was also demonstrated using WGA, which can bind to cell walls of selected *Listeria* strains [20]. Recognition and binding of WGA requires non-reducing, terminal GlcNAc residues [31], such as those present on *Listeria* cells of serovars 1/2 and 3 WTAs, but not on the mutant strain WSLC 1442 [27]. Lack of GlcNAc in WSLC 1442 WTA as shown by ESI-MS/MS is responsible for the failure to bind the lectin. WGA also fails to bind to WSLC 1042 serovar 4 cells, where GlcNAc is not only an integral component of the poly(RboP) chain, but may be further decorated by hexose moieties such as Glc and Gal. Binding of carbohydrate-specific lectins can be exploited to determine principal presence of terminal GlcNAc on polymers such as WTAs. Similarly, highly specific cell wall binding domains of bacteriophage endolysins [20] or cell wall recognition properties of phages [27], [32], [33] can be used to examine WTA glycosylation among bacteria where these abundant molecules serve as the primary binding ligands (Eugster *et al.*, manuscript submitted for publication). However, care must be taken using these functional assays, since failure to bind does not necessarily indicate lack of the ligand, but may be due to masking or modification, or changes in expression or quantity of these molecules.

In conclusion, we here describe a simple, rapid, sensitive and specific method for analyzing WTAs from bacterial cell walls by ESI-MS/MS. It allows analysis of monomer units of WTAs, and does not require total chemical degradation of WTA into its components, and calculation of molar ratios of substituents and the backbone. The method gathers most of the information needed for principal WTA component analysis to evaluate similarities and differences in WTAs between bacterial strains, even from bacteria with a complex WTA structure. It is not limited by complexity, molecular size and length of different WTA polymers since monomers are analyzed directly after acid hydrolysis without additional purification. We expect that this approach will facilitate structural characterization and analysis of cell wall carbohydrate polymers from diverse bacterial species and genera.

## Materials and Methods

### Bacterial strains and growth conditions

Bacteria used in this study were *L. monocytogenes* strains EGDe (clinical isolate, serovar 1/2a), strain WSLC 1442 (serovar 1/2a, lacking GlcNAc in WTA [27]), strain WSLC 1485 (serovar 3a), and strain WSLC 1042 (serovar 4b). Bacteria were cultured at 30°C in tryptose broth (TB) with shaking as described previously [34].

### Preparation and purification of cell walls

The walls from *L. monocytogenes* cells were prepared similar to the methods described previously [13], [27], [35]. Cells of exponentially growing cultures (A_600_ of 0.7 to 0.8) were heat-killed by steaming for 30 min, harvested by centrifugation (7,000× *g*, 15 min, 10°C), resuspended in SM buffer (100 mM NaCl, 8 mM MgSO_4_, 50 mM Tris-HCl, pH 7.5), and frozen overnight at −20°C. Thawed cells were disrupted by double passage through a One-Shot system (Constant Cell Disruption System, Northants, UK) at a pressure of 270 MPa. After removing unbroken cells and debris by centrifugation at 1,400× *g* for 5 min, cell wall material was recovered by centrifugation (20,000× *g*, 30 min, 4°C), washed three times with water, and resuspended in SM buffer. The resulting fraction was treated with DNase/RNase at room temperature for 3.5 h, and incubated with proteinase K for another 2 h with gentle mixing to remove proteins (each enzyme at a final concentration of 100 µg per gram wet crude cell walls). Cell walls were then extracted with boiling 4% (w/v) SDS solution for 30 min. Insoluble material was recovered by centrifugation (20,000× *g*, 30 min, 20°C) and washed 5 times with sterile deionized water by repeated centrifugation and resuspension. The procedure yielded carbohydrate cell wall material devoid of proteins and lipids. The material was lyophilized and stored in water at −20°C until use.

### Extraction of wall teichoic acids

To extract WTAs from cell walls, lyophilized cell walls were treated with 25 mM glycine/HCl buffer (pH 2.5) at 100°C for 10 min [14], [27], [36], [37]. Insoluble material was removed from extracted soluble WTAs by centrifugation at 30,000× *g* for 30 min and resuspended in the same buffer. The extraction was repeated twice, and pooled supernatants containing the extracted WTA material were then dialyzed (MWCO 1,000) against purified deionized water at 4°C, with three changes of water. The resulting crude WTA fraction was again lyophilized for storage.

### Purification of wall teichoic acids

WTAs from the crude preparations were purified by anion exchange chromatography, as reported earlier [13], [27]. Briefly, 10 mg crude WTAs dissolved in 1 ml starting buffer (10 mM Tris-HCl, pH 7.5) were applied to a HiTrap DEAE FF column (5 ml; GE Healthcare, Glattbrugg, Switzerland) previously equilibrated with the same buffer. The column was eluted with a linear gradient (approximately 20 column volumes) of 0 to 1 M NaCl in 10 mM Tris-HCl (pH 7.5) at 20°C. Fractions were collected at a flow rate of 1 ml/min, and monitored using a UV detector set to 205 nm wavelength.

### Phosphate determination for teichoic acid quantification

The WTA content in various chromatography fractions was quantified as total phosphate using a combination of two methods. For oxidative decomposition, 30 µl fractions were diluted to a volume of 5 ml with distilled water, and treated with a decomposition reagent (NANOCOLOR NanOx Metal; Macherey-Nagel, Oensingen, Switzerland) according to the manufacturer's instructions. After pretreatment, total phosphorus was determined photometrically using a phosphate test kit (Spectroquant® Phosphate Test; Merck, Zug, Switzerland). The calibration curve for quantitative measurement was established with aqueous dilutions of a 1,000 mg/l phosphate standard solution (VWR, Dietikon, Switzerland) in the range of 0.1 to 5.0 mg/l inorganic phosphate. The reaction of phosphate ions and molybdate to molybdophosphoric acid and its subsequent reduction to phosphomolybdenum blue was monitored at 690 nm in standard 10 mm polystyrene cuvettes, after incubation for 10 min. Pure water was used as a blank. Phosphorus-containing (i.e., WTA containing) fractions were pooled, dialyzed against distilled water (three changes of water, 4°C), and lyophilized.

### Acid hydrolysis of wall teichoic acids polymers

Selective cleavage of phosphodiester linkages in WTA polymers was achieved by treatment with 48% HF, as described earlier [19], [38], [39]. Aliquots of 1 mg lyophilized WTA was added to 200 µl 48% HF, and incubated in ice (0°C) for 16 h. Following removal of HF by evaporation over NaOH pellets under vacuum in a glass-free system, WTA monomers were stored in pure water at −20°C, or lyophilized for further analysis.

### Mass spectrometry analysis

ESI-MS/MS analysis of WTA monomers was performed at the Functional Genomics Center Zurich (FGCZ) of ETH Zurich and the University of Zurich (Switzerland), using a Q-TOF Ultima API mass spectrometer (Micromass, UK) equipped with an electrospray ion source. Samples were measured in the positive ion mode.

For analysis of WTAs from strains WSLC 1442 and WSLC 1042, lyophilized WTA monomer samples were dissolved in a small volume of acetonitrile containing 3 mM lithium trifluoroacetate (Li-TFA, added from a 5 mM stock solution in H_2_O). No additive was used for the measurement of WTAs of strains EGDe and WSLC 1485. The samples were dissolved in 0.2% formic acid in 50% acetonitrile prior to measurement by ESI-MS and ESI-MS/MS. For MS/MS, argon was used as the collision gas for fragmentation of parent ions. The resulting fragment ions were separated by a time-of-flight (TOF) mass analyzer.

### Binding assay and fluorescence microscopy

A fluorescent WGA-Alexa Fluor® 594 conjugate (Invitrogen, Basel, Switzerland) was used to detect terminal GlcNAc residues on *Listeria* WTA [20], [31]. For this purpose, *Listeria* cells were harvested from late log phase cultures by centrifugation at 7,000× *g* for 60 s and resuspended in 1/10 volume PBST. One-hundred µl samples were mixed with 50 µl of WGA solution (0.1 mg/ml), and incubated for 5 min at room temperature. Bacterial cells with bound lectin were recovered by centrifugation at 16,000× *g* for 1 min, and washed twice in 500 µl of PBST. The pellet was resuspended in 50 µl of PBST buffer and observed using confocal laser scanning microscopy (Leica TCS SPE; Leica, Heerbrugg, Switzerland).
